# Significant Decreased Expressions of CaN, VEGF, SLC39A6 and SFRP1 in MDA-MB-231 Xenograft Breast Tumor Mice Treated with *Moringa oleifera* Leaves and Seed Residue (MOLSr) Extracts

**DOI:** 10.3390/nu12102993

**Published:** 2020-09-30

**Authors:** Wai Feng Lim, Mohd Izwan Mohamad Yusof, Lay Kek Teh, Mohd Zaki Salleh

**Affiliations:** 1 Integrative Pharmacogenomics Institute (iPROMISE), Universiti Teknologi MARA, Puncak Alam Campus, Bandar Puncak Alam 42300, Selangor Darul Ehsan, Malaysia; limwaifeng85@gmail.com (W.F.L.); mohd_izwan86@yahoo.com (M.I.M.Y.); 2 Faculty of Pharmacy, Universiti Teknologi MARA, Puncak Alam Campus, Bandar Puncak Alam 42300, Selangor Darul Ehsan, Malaysia

**Keywords:** moringa leaves and seed residue, chemoprevention, MCF-7, MDA-MB-231, gene expression profiling

## Abstract

*Moringa oleifera* is a miracle plant with many nutritional and medicinal properties. Chemopreventive values of the combined mixture of moringa leaves and seed residue (MOLSr) at different ratios (M1S9, M1S1 and M9S1) were investigated. MOLSr extracts were subjected to phytochemical screening, antioxidant assays, metabolite profiling and cytotoxic activity on the primary mammary epithelial cells (PMECs), non-malignant Chang’s liver cells and various human cancer cell lines (including breast, cervical, colon and liver cancer cell lines). The MOLSr ratio with the most potent cytotoxic activity was used in xenograft mice injected with MDA-MB-231 cells for in vivo tumorigenicity study as well as further protein and gene expression studies. M1S9, specifically composed of saponin and amino acid, retained the lowest antioxidant activity but the highest glucosinolate content as compared to other ratios. Cell viability decreased significantly in MCF-7 breast cancer cells and PMECs after treatment with M1S9. Solid tumor from MDA-MB-231 xenograft mice was inhibited by up to 64.5% at third week after treatment with high-dose M1S9. High-dose M1S9 significantly decreased the expression of calcineurin (CaN) and vascular endothelial cell growth factor (VEGF) proteins as well as the secreted frizzled-related protein 1 (SFRP1) and solute carrier family 39 member 6 (SLC39A6) genes. This study provides new scientific evidence for the chemoprevention potential of MOLSr extracts in a breast cancer model; however, the precise mechanism warrants further investigation.

## 1. Introduction

According to the World Health Organization (WHO), cancers of the lung, colon, stomach, liver and breast contributed to approximately 9.6 million of global deaths in 2018 [1]. Cancer is characterized by abnormal cell growth that invades and spreads to other parts of the body (called metastasis). Although chemotherapy is a widely used treatment for cancer, the challenge remains due to multi-drug resistance of chemotherapy leading to ineffective outcomes [2]. The old adage applies—”prevention is better than cure”. The scientific community is actively venturing into cancer prevention, i.e., chemoprevention, to minimize and alleviate these dreaded effects and complications of cancer and conventional chemotherapy. 

Chemoprevention modulates specific pathways in carcinogenesis including cell proliferation, invasion and metastasis, immune destruction, apoptosis and angiogenesis to reverse, suppress or prevent cancer progression to invasive cancer by using natural, synthetic or biological agents [3,4,5,6]. It is desirable to incorporate a chemoprevention strategy as part of precision health to improve prognosis with agents which have milder and negligible side effects in cancer patients. However, more work is required to realize this. Plants with anti-cancer properties have been investigated as potential sources of chemopreventive agents. This includes *Moringa oleifera* Lam. (*M. oleifera*) [7,8]. Moringa (MO) is a plant family, Moringaceae, mostly found in tropical and subtropical regions, with at least 13 species. Various parts of *M. oleifera*, including leaves, seed, root and bark extracts, have been reported to possess anti-cancer activities by interfering in the oncogenesis process, cancer cell growth and progression in several types of cancer including breast cancer, colorectal cancer and liver cancer [9,10,11,12]. 

MO is classified under the order of Brassicales, predominantly with the abundance of glucosinolates [7,13]. Moringa-derived glucosinolates possess chemoprotective potential through cytoprotective, anticarcinogenic and antioxidant effects and induces apoptosis in cancer cells [14,15]. In intact plants, glucosinolates are secondary metabolites of phytochemicals that are mainly found in moringa leaves (MOL) and moringa seeds (MOS), with seeds having 8% higher glucosinolate levels as compared to other parts of the moringa plant [7,16]. MOS, which has up to 40% oil and high-quality fatty acid composition, is extensively used for human consumption and commercial purposes like cosmetics, biodiesel and others [17,18]. A part remains after oil seed extraction (with minimal production costs), moringa seed residue (MOSr), also known as press cake, seed cake or cake residue, which retains mainly the positively charged protein [19]. MOSr serves as a scavenger for heavy metal removal, as a natural coagulant for wastewater treatment and has also been found to possess antibacterial properties [20,21]. 

Aqueous/soluble MOL extracts were shown to inhibit cancer cell proliferation and induce apoptosis to combat tumor progression in human tumor (KB) cancer cells and human lung A549 cancer cells [22,23]. The hot water MOL extracts exhibited anti-proliferative potential in A549 lung cancer and esophageal cancer cells by upregulating the apoptotic markers leading to cancer cell death [24]. As compared to normal BHK-21 cells, ethanolic moringa root core extracts stimulated three-fold more effective cell death and triggered higher apoptotic events in colorectal HCT 116/Caco-2 cancer cells [10]. After treatment with ethanolic MOL extracts and bark extracts, both the breast MDA-MB-231 and colorectal HCT-8 cancer cells showed reduced surviving cells, increased apoptotic cells, arrested cell cycles and displayed remarkable phenotypic changes. However, treatment with ethanolic MOS extracts showed no noticeable changes. These phenomena demonstrated that the anti-cancer effect of moringa was exhibited only by its leaf and bark but not the seed extracts [9]. The ethanolic MOS extracts showed no remarkable anti-cancer properties in both breast MDA-MB-231 and colorectal HCT-8 cancer cells [25]. On the contrary, water-, hexane- and dichloromethane-extracted MOS inhibited the growth of MCF-7 breast cancer cells, with only hexane-fractionated MOS extracts exerting minimal toxic effects on normal MCF-10A cells [11]. Different findings on the efficacy of MO extracts reported thus far are mostly due to the different solvents used for extraction, leading to variation in the phytochemical compositions of the extracts and subsequently the plant’s medicinal properties. Furthermore, the phytochemical compositions of plant extracts are not only influenced by the extraction methods and solvents used but also vary according to ecological, evolutionary and geographical factors [26].

Since the extracts of MOL and MOSr possess different chemical constituents, we believed that by combining the extracts, a wider spectrum of activities would be observed. Therefore, in this study, we used a combination of MOL and MOSr extracts. MOS extracts possess 8% higher glucosinolates than leaves, where glucosinolates have been claimed to possess chemopreventive potential while the MOL extracts have shown good antioxidant activities and other nutritional values which are not reported in the seed [7,8,12,14,16]. The combination extracts were first investigated for their phytochemical composition, antioxidant activity and metabolite profiles. Next, the biological effects of the combination extracts were examined using cell lines (in vitro model) and the ratio of MOL and MOSr extracts which resulted in the highest cytotoxic effect was determined. We subsequently investigated the chemopreventive properties of the MOLSr extracts using an in vivo model to delineate the mechanistic pathways. 

## 2. Materials and Methods 

### 2.1. Preparation of Moringa Mixtures

*M. oleifera* leaves (MOL) and seed residue (MOSr) were obtained from the Borneo Moringa Sdn. Bhd. (Malaysia) in Tenom, Sabah, Malaysia with voucher identification number Bm_mo_191012_1. The ethanolic extracts of the MOL and MOSr were provided by MitoMasa Sdn. Bhd. in freeze-dried format. Three combinations of different ratios of the MOL and MOSr extract were freshly prepared in water, with the final concentration of 10 mg/mL. M9S1 consisted of 9 mg of MOL extract and 1 mg of MOSr extract. M1S1 consisted of 5 mg of MOL extract and 5 mg of MOSr extract. M1S9 consisted of 1 mg of MOL extract and 9 mg of MOSr extract. Figure S1 shows the experimental design for this study. 

### 2.2. Phytochemical Screening, Total Flavonoid, Phenolic Content and Antioxidant Assays

Moringa mixtures were subjected to phytochemical analysis following methods described by Harborne (1998) and Kokate (2005), which are based on visual observation of color changes or the formation of precipitates [27,28]. Quantitative analysis of total flavonoid content (TFC) and total phenolic content (TPC) of moringa mixtures were determined spectrophotometrically by the aluminum calorimetric method (quercetin as the reference standard) and Folin–Ciocalteu method (gallic acid as the reference standard) with minor modifications, respectively. The antioxidant capacity of moringa mixtures was tested using 2,2-diphenyl-1-picrylhydrazyl (DPPH) free radical-scavenging assay (Trolox as the reference standard) and ferric reducing antioxidant power (FRAP) assay (Trolox and ascorbic acid as the reference standard and positive control, respectively). These antioxidant assays were carried out according to Yang et al. (2011) with slight modifications [29]. Detailed steps were described previously by Abdul Hisam et al. (2018) [30]. All the experiments were conducted in triplicate.

### 2.3. Metabolite Profiling

Liquid chromatography mass spectrometry/quadrupole time of flight (LC-MS/QTOF) was used to identify bioactive compounds in extracts of moringa leaves (MOL), moringa seed residue (MOSr) and moringa mixtures at different ratios: M1S9, M1S1 and M9S1. The stock solutions were purified by a solid phase extraction (SPE) procedure. Two microliters of each sample were injected and analyzed by LCMS-QTOF (model 6520 Agilent Technologies, Santa Clara, CA, USA). The chemical entities of each sample were resolved using a ZORBAX Eclipse Plus C18 column (100 mm × 2.1 mm × 1.8 µm, Agilent Technologies, Santa Clara, CA, USA) maintained at 40 °C. The flow rate of 0.25 mL/min with solvent A (water with 0.1% formic acid) and solvent B (acetonitrile with 0.1% formic acid) was used. A linear gradient was developed over 36 min from 5% to 95% of mobile phase (B). The total run time was 48 min for each analysis. Electrospray ionization (ESI) source was set with a V Cap 4000 V, skimmer 65 V and fragmentor 125 V. The nebulizer was set at 45 psig and the nitrogen drying gas was set at a flow rate of 12 L/min. The drying gas temperature was maintained at 350 °C. Data were collected in positive ESI ionization mode and in full scan mode from 100 to 1000 *m/z*. During the analysis, two reference masses of 121.0509 *m/z* (C_5_H_4_N_4_) and 922.0098 *m/z* (C_18_H_18_O_6_N_3_P_3_F_24_) were continuously injected to allow correction for accurate mass.

### 2.4. Cell Culture Growth Conditions 

Human primary mammary epithelial cells (PMECs) (ATCC^®^ PCS 600-010™) were purchased from ATCC (American Type Culture Collection, Manassas, VA, USA) and maintained in mammary epithelial cell basal medium (ATCC^®^ PCS-600-030™) supplemented with mammary epithelial cell growth kit (ATCC^®^ PCS-600-040™) and incubated at 37 °C in a humidified 5% CO_2_ atmosphere according to the manufacturer’s guidelines. Non-malignant Chang’s liver (ATCC^®^ CCL-13), human hepatocellular carcinoma (HepG2) (ATCC^®^ HB-8065™), cervical adenocarcinoma (HeLa) (ATCC^®^ CCL-2™) and human breast adenocarcinoma (MCF-7) (ATCC^®^ HTB-22™) cell lines were maintained in Eagle’s minimum essential medium (ATCC^®^ 30-2003™), respectively (Sigma Aldrich, St Louis, MO, USA). Human colorectal carcinoma (HCT-116) cell lines (ATCC^®^ CCL-247™) were cultured in McCoy’s 5A (ATCC^®^ 30-2007™). These cultures were supplemented with 10% fetal bovine serum (Biowest, Kansas City, MO, USA) and incubated at 37 °C in a humidified 5% CO_2_ atmosphere. When the cells reached 70–80% confluency, the cells were detached with phosphate buffer saline (Sigma Aldrich, St Louis, MO, USA), trypsinized with 3 mL of trypsin-EDTA (Nacalai Tesque, Kyoto, Japan) and either seeded in a 96-well plate or sub-cultured.

### 2.5. Cell Viability Assessment 

The 3-(4,5-dimethylthiazol-2-yl)-2,5-diphenyltetrazolium bromide (MTT) assay is a mitochondrial-based cytotoxic test on cell viability. A total of 1 × 10^5^ of cells were seeded overnight and then treated with tamoxifen (TAM) (positive control) and moringa mixtures (M1S9, M1S1 and M9S1) with two-fold series dilutions from 100 to 6.25 µg/mL, at three time points (24, 48 and 72 h), respectively. At each time point, 20 µL of 5 mg/mL dimethyl-thiazole-tetrazolium (MTT) (Sigma Aldrich, St Louis, MO, USA) in phosphate buffer (Sigma Aldrich, St Louis, MO, USA) was added and further incubated at 37 °C for three hours. The crystallized MTT was solubilized with DMSO before the concentration was determined by optical density at 570 nm using a microplate reader (BMG POLARstar Omega, Ortenberg, Germany). Wells with non-treated cells and only DMSO solution served as negative (100% cell viability) and background (for blank subtraction) controls, respectively. The percentages of viable cells were plotted against concentrations to determine cell viability (%) and the IC_50_ value. All experiments were conducted in triplicate.

### 2.6. Xenograft Mice Model

Animal study was approved by the UiTM Committee on Animal Research and Ethics (Ethic approval number: UiTM CARE 278/2019 (05/04/2019)). All animal experiments were conducted in accordance with standard ethical guidelines. Six- to eight-week-old female SPF NOD/ShiJic-SCID mice were obtained from Nomura Siam International (Bangkok, Thailand). All mice were housed in the pathogen-free environment under controlled conditions (temperature 20–26 °C, humidity 40–70%, light/dark cycle 12 h/12 h) and received water and food ad libitium. To evaluate the tumorigenesis in the animal model, the highly aggressive MDA-MB-231 cell lines were injected into SCID mice to induce the breast cancer model. MDA-MB-231 cell lines (ATCC^®^ HTB-26™) were grown in Leibovitz’s medium (L15) (HyClone, Rd. Logan, UT, USA) with 10% fetal bovine serum (FBS) (Gibco, Gaithersburg, MD, USA) and 1% penicillin-streptomycin (Beyotime Biotechnology, Haimen, Jiangsu, China) in a humidified incubator at 37 °C without CO_2_. Mice were subcutaneously injected with 3 × 10^6^/100 μL MDA-MB-231 cells into the right flank region in the SCID mice. 

### 2.7. In Vivo Tumorigenicity Study

After tumor volume had reached 100–150 mm^3^, the xenograft mice were randomized into five groups (n = 6) and received normal saline orally (negative control), M1S9 orally (250 mg/kg, 500 mg/kg, 750 mg/kg) and 30 mg/kg TAM subcutaneously (positive control) for 60 consecutive days or until the reduction in tumor size was 40–60% (Figure S2). Dose and route used for positive control (tamoxifen 30 mg/kg) were based on the method described by Yoneya et al. (2010) [31]. Body weight and tumor volume were measured weekly up to two months. The tumor-growth-inhibition rate (I.R.) was calculated using the following formula: I.R. (%) = {[(Average tumor volume of the control − average tumor volume of the treated group)/Average tumor volume of the control] * 100%}. After two months, serum blood was collected for biochemical analysis. Subsequently, all organs (liver, kidney, spleen, heart and lung) and tumors were excised, weighted and calculated as relative organ weight (expressed as the percentage of organ weight to body weight). The tumors were snap-frozen in liquid nitrogen for gene expression analysis later.

### 2.8. Protein and Gene Expression Analysis 

Serum blood was subjected to biochemical analysis on calcineurin (CaN), estrogen (ES) and vascular endothelial cell growth factor (VEGF) proteins using ELISA kits (FineTest, Wuhan, China), following the manufacturer’s instructions. The Human Breast Cancer RT^2^ Profiler PCR array (Rotor-Gene^®^ Format) (PAHS-131ZR, Qiagen^®^ GmbH, Hilden, Germany) was used to profile the expression of 84 genes associated with key human breast cancer pathways. Briefly, total RNA was isolated from pulverized tumor tissues of non-treated (negative control), high-dose M1S9-treated mice and TAM-treated (positive control) using RNeasy^®^ Lipid Tissue Mini kit (Qiagen^®^ GmbH, Hilden, Germany). Three biological replicates were prepared from each group. Eluted RNA was quantitated and purity-checked using UV-absorbance-based NanoDrop^®^ (Thermo Scientific, Wilmington, NC, USA) and Agilent 2100 Bioanalyzer RNA 6000 Nano Chip (Agilent, Palo Alto, CA, USA), respectively. A total of 1000 ng of RNA sample was converted to cDNA using RT^2^ First Strand Kit (Qiagen^®^ GmbH, Hilden, Germany) according to the manufacturer’s protocol. The cDNA was then mixed with RT^2^ SYBR Green ROX FAST Mastermix and RNase-free water before dispensing into the array and then subjected to Rotor-Gene^®^ Q real time PCR cycler (Qiagen^®^ GmbH, Hilden, Germany) for analysis. Threshold cycle (C_T_) of each gene was analyzed for ΔΔC_T_ after normalization using housekeeping genes in the web-based RT^2^ Profiler Data Analysis Software (https://www.qiagen.com/my/shop/genes-and-pathways/data-analysis-center-overview-page/). Gene expression was considered significantly regulated by TAM and M1S9 when the mean fold regulation was greater than ±2.

### 2.9. Gene Function and Gene Network Analysis

The web-based Database for Annotation, Visualization and Integrated Discovery (DAVID) gene annotation tool (https://david.ncifcrf.gov) was used to enrich the up- and downregulated genes (fold regulation more than ±2) to understand the biological effects (mechanism) of TAM and M1S9 towards MDA-MB-231 tumors. Gene ontology (GO) and Kyoto Encyclopedia of Genes and Genomes (KEGG) were used to classify those genes based on their cellular functions and biological pathways involved. The Homo sapiens database was selected as the background in the enrichment analysis. The GO biological process and KEGG pathway enrichment analyses were considered significant with *p*-value less than 0.05 and enrichment gene count greater than or equal to three. Subsequently, gene network analysis was performed using Qiagen’s Ingenuity Pathway Analysis (IPA, Qiagen Redwood City, CA, USA) software. IPA database contains all known interactions and was used to elucidate the biological interpretation of genes in regulating the MDA-MB-231 cells with TAM and M1S9 extracts.

### 2.10. Statistical Analysis

The data were expressed as the mean ± SEM values (n = 3), using one-way ANOVA followed by Dunnett’s *t*-test (*p* < 0.05). All statistical analyses were performed using statistical software package, IBM^®^ SPSS^®^ Statistics Version 22.

## 3. Results

### 3.1. M1S9 Extracts Detected the Presence of Saponins, Amino Acids and High Glucosinolate Content but with Low Antioxidant Activity

The qualitative phytochemical screening revealed the presence of tannins, flavonoids, phenols, proteins, fats and fixed oils as well as carbohydrates in M1S1 and M9S1 (Table 1). However, flavonoid was not detected in M1S9 but saponins and amino acids were detected instead. M1S9 extracts possessed the lowest level of total flavonoid content (TFC), total phenolic content (TPC) and antioxidant activity compared to other ratios. For metabolite profiling (Figure S3), a total of 17 compounds were identified across different extracts’ compositions (Table 2). Compounds found uniquely in MOL extracts included flavonoids (such as isoquercetin, quercetin, astragalin, isorhamnetin and kaempferol) while in MOSr, glucosinolates (glucosinalbin and glucotropaeolin), glycoside (niazirin), phenolic acid (p-coumaric acid) and other naturally occurring compounds (benzoic acid, vanillin and heptadecane) were identified. Although other studies showed that glucosinolates are predominantly found in both MOL and MOSr extracts, this study found no glucosinolates in the MOL extracts, mainly influenced by the different preparation method [32].

### 3.2. M1S9 Extracts Exhibited the Cytotoxic Effect on MCF-7 Breast Cancer Cell Line

Among all the tested cell lines, the IC_50_ values of tamoxifen (TAM) (serves as positive control) were relatively low, ranging from 3 to 15.5 μg/mL; it reduced cell viability not only in normal cells but also in cancer cell lines in a dose- and time-dependent manner (Table 3). Once characterized in PMECs, M1S9 and M1S1 showed toxic effects towards these cells while no obvious cytotoxic effect was observed in M9S1-treated cells. M1S9 extract exhibited the highest reduction in MCF-7 breast cancer cell viability at IC_50_ values equal to 38.5 μg/mL (72 h). A previous study found that MCF-7 cells were less tumorigenic as they are slow growing primary tumors in the mammary fat pad and no metastases were observed in a mouse model [33]. Therefore, the highly aggressive MDA-MB-231 cells were injected into SCID mice to establish the in vivo model for elucidation of the anti-cancer effects using M1S9 extracts. 

### 3.3. High-Dose M1S9 Extracts Suppressed Tumor Growth in MDA-MB-231 Xenograft Tumor

**Figure 1:**
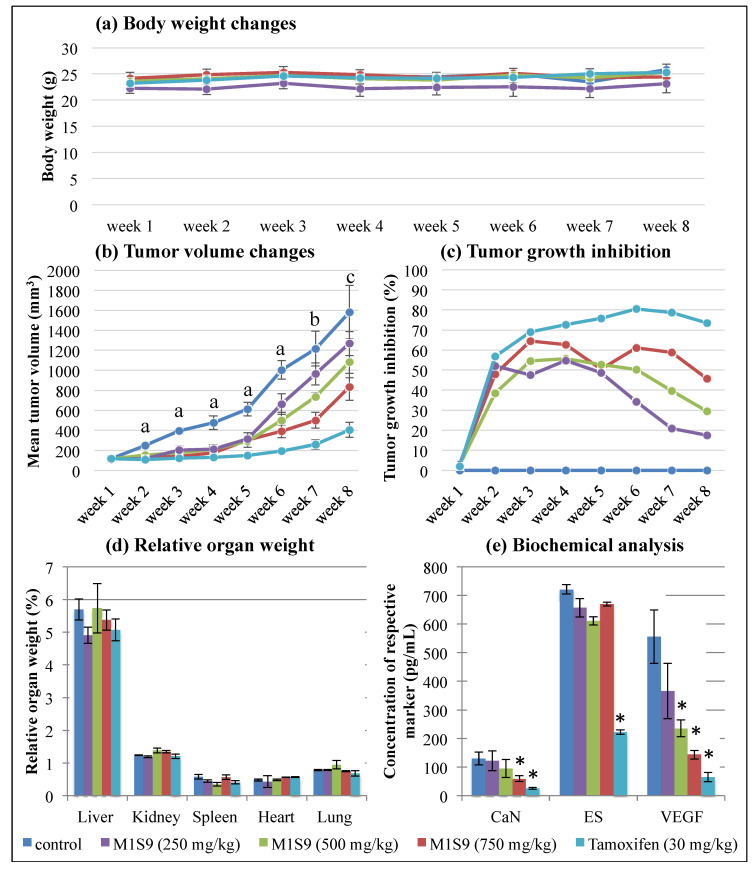
The effects of M1S9 extracts on breast tumor growth using MDA-MB-231 xenograft animal model: (**a**) body weight changes (g); (**b**) tumor volume changes (mm^3^); (**c**) tumor growth inhibition (%); (**d**) relative organ weight (%); (**e**) biochemical analysis (pg/mL) over eight weeks after treatments with three doses of M1S9 extracts, normal saline (negative control) and tamoxifen 30 mg/kg (positive control). Values are in mean ± S.E.M. In tumor volume changes, a, b and c indicate significant differences (*p* < 0.05) with the control at the same week as determined by Dunnett test, a non-parametric test. “a” was significant difference in all treatment groups, “b” was significant difference in all treatment groups except low dose M1S9 (250 mg/kg) and “c” was significant difference in all treatment groups except low dose M1S9 (250 mg/kg) and medium dose M1S9 (500 mg/kg). In biochemical analysis, asterisk (*) means significant difference from control group (*p* < 0.05). Abbreviations: CaN—calcineurin; ES—estrogen; VEGF—vascular endothelial cell growth factor.

At the end of M1S9 treatment, no significant difference in body weight changes was observed in xenograft mice treated with M1S9 and TAM up to eight weeks (Figure 1a). However, the tumor volume of MDA-MB-231 showed a significant reduction in mice treated with M1S9 (all three doses) and TAM, compared to the control mice (*p* < 0.05), up to six weeks (Figure 1b). Referring to Figure 1b, high-dose M1S9 extract was able to inhibit tumor growth and the inhibitory effects were sustained throughout the experimental period, while low-dose and medium-dose M1S9 extracts were not able to inhibit tumor growth effectively (no significant data shown) after the sixth week and seventh week, respectively. The maximum percentages of tumor growth inhibition were 64.5% for high dose of M1S9 extract (at week 3), 55.6% for moderate dose of M1S9 extract (at week 4) and 54.8% for low dose of M1S9 extract (at week 4), respectively (Figure 1c). The relative organ weights for the liver, heart, spleen, lung and kidney were unaffected by different dosages of M1S9 extracts and TAM (*p* > 0.05) when compared to the control group (Figure 1d), suggesting that the extracts did not induce any toxic effect on these organs [34,35].

### 3.4. High-Dose M1S9 Extracts Downregulated the Expression of CaN and VEGF Proteins as Well as SFRP1 and SLC39A6 Genes in MDA-MB-231 Xenograft Tumor

**Figure 2:**
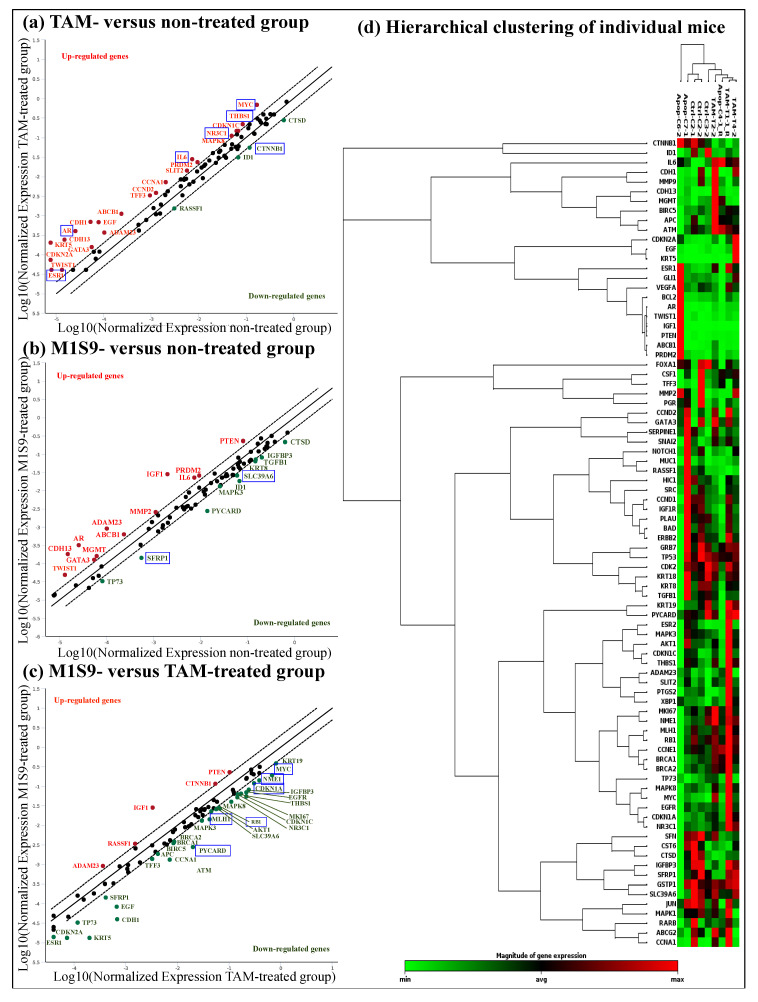
Effect of M1S9 on the expression levels of 84 key human breast cancer genes in MDA-MB-231 xenograft tumor: (**a**) TAM-treated versus non-treated group; (**b**) M1S9-treated versus non-treated group; (**c**) M1S9-treated versus TAM-treated group; (**d**) hierarchical clustering of individual mice. Upregulated genes (red characters) and downregulated genes (green characters) have fold changes ≥ ±2. Blue bracket indicates genes with significant difference at *p* < 0.05. Abbreviation: TAM—tamoxifen.

The biological effects mediated by high-dose M1S9 over the non-treated groups were investigated at the selected protein level (Figure 1e) and transcript level (Figure 2). Both CaN and VEGF levels showed significant reductions in the mice treated with high-dose M1S9 while in TAM-treated cells, CaN, ES and VEGF protein levels were decreased significantly compared with corresponding non-treated xenograft mice (control). Interestingly, the VEGF levels showed a dose-dependent decrease after being treated with M1S9. For the gene expression study, AR, ESR1, IL6, MYC, NR3C1 and THBS1 genes were induced significantly (*p* < 0.05) and only CTNNB1 gene was suppressed significantly (*p* < 0.05), respectively, in TAM-treated cells. After M1S9 treatment, only two genes showed downregulation with significant differences, including SFRP1 and SLC39A6. An overview of the functional role of each differential expressed gene is presented in Table S1. The TAM-treated group participated actively in steroid receptor-mediated, glucocorticoid and classical WNT signaling. Significantly downregulated CTNNB1 gene was involved in epithelial-to-mesenchymal transition (EMT), angiogenesis and cell adhesion molecule processes. For M1S9-treated group, only classical WNT signaling and the apoptosis process were significantly regulated by the SFRP1 gene.

### 3.5. High-Dose M1S9 Regulated Genes Enriched in Specific Biological Processes, Pathways and Gene Networks

**Figure 3:**
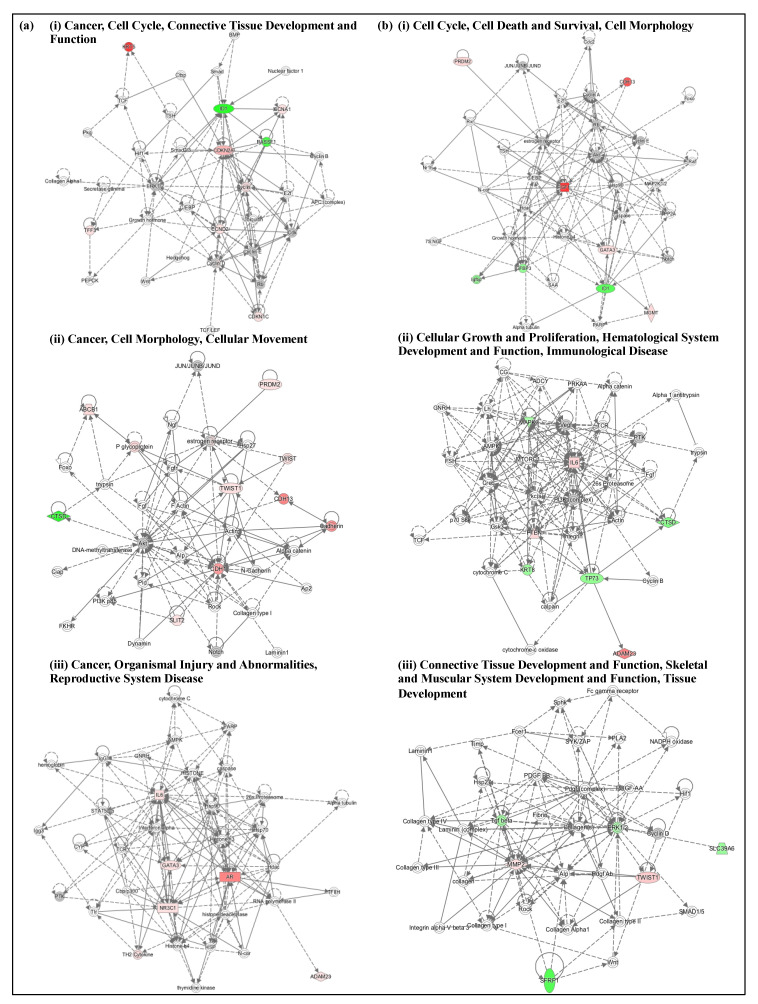
Top three enriched networks in (**a**) TAM-treated; (**b**) M1S9-treated MDA-MB-231 xenograft tumor. Up/downregulated genes (fold changes ≥ ±2) are colored in red and green, respectively, while the color intensities are relative to fold change. Nodes without color were neither expressed nor assessed in this study. Abbreviation: TAM—tamoxifen.

TAM-treated and high-dose M1S9-treated groups were significantly enriched in the biological transcriptional regulation processes, which are important to understand the role of TAM and M1S9 in the pathogenesis of breast cancer (Tables S2–S4). GO terms were enriched specifically in the M1S9-treated group including regulation of MAPK, ERK1 and ERK2 cascade, response to ethanol, viral process and protein phosphorylation. For KEGG pathway enrichment analysis, both treatments participated in pathways of cancer, proteoglycans in cancer and several signaling pathways including P13K-Akt, FoxO and Hippo. Gene networks were built to illustrate the correlations between differentially expressed genes (Figure 3, Table S5). For M1S9-treated MDA-MB-231 cells, related diseases and functions that overlapped with TAM-treated cells were cell cycle, cell morphology and connective tissue development and function. However, unique diseases and functions related to M1S9-treated cells were cell death and survival, cellular growth and proliferation, hematological system development and function, immunological disease, skeletal and muscular system development and function as well as tissue development. 

## 4. Discussion

All the different parts of the moringa (MO) plant contain valuable compounds with anti-cancer activity as reported by ethanolic extracts of moringa leaves (MOL) and moringa seed (MOS) [9,10,11,12]. We further studied the anti-cancer potential of different combination ratios of moringa leaves (MOL) and moringa seed residue (MOSr) (after oil repletion with minimal production costs) extracts. In the phytochemical analysis, the specific bioactive compounds in M1S9 included saponins and amino acids but were not found in other combinations with higher ratios of leaf extracts, suggesting that these compounds were mainly derived from the MOSr extracts. A previous study showed that *Camellia oleifera* and *Camelina sativa* seed cakes (herein referred to as seed residue) have a specific group of saponin compounds, i.e., triterpenoid saponins, that possess anti-tumor activity [36]. After oil repletion, the MOSr still retain the nutritive properties of amino acids, which were only reported in moringa seeds (MOS) but not in MOL [37]. A recent study showed that water extracts of *M. oleifera* seed kernel possess potent antioxidant activity due to high total phenolic, flavonoid and tannin contents [38]. However, antioxidant activity of MOS extract had diminished after oil repletion, probably related to low flavonoid and phenolic contents. Glucosinalbin (a glucosinolate) was the most abundantly found compound in MOSr extract, in which MOS extract had an 8% higher glucosinolate level as compared to other parts of moringa [7,16]. Moringa-derived glucosinolates possess chemoprotective potential through cytoprotective, anticarcinogenic and antioxidant effects and induced apoptosis in cancer cells [14]. Therefore, the combined mixture of MOL and MOSr extracts could have demonstrated chemopreventive properties via biological processes other than antioxidant processes.

According to the American National Cancer Institute guidelines for plant screening, in vitro cytotoxic tests of any extracts that have IC_50_ values equal to or less than 20 μg/mL are considered to have promising anti-cancer potential [39]. M1S9 exerted a profound suppressive effect on the cell viability of the MCF-7 cells (IC_50_ = 38.5 μg/mL) as well as the PMECs (the normal breast cells) (IC_50_ = 17 μg/mL) after 72 h of treatment. Araújo et al. (2013) had determined that the aqueous extract of MOS was potentially cytotoxic to human peripheral blood mononuclear cells (IC_50_ = 34.3 µg/mL) [40]. This might be due to the presence of isothiocyanate (ITC) in the MOS extract that is toxic to the normal cells [41]. The ethanolic extracts of MOL and bark exhibited anti-cancer properties while MOS extract showed no remarkable anti-cancer properties in both breast MDA-MB-231 and colorectal HCT-8 cancer cells [9,25]. On the other hand, the dichloromethane fraction of the MOS extract was highly toxic to MCF-7 (IC_50_ = 26 µg/mL) but had limited toxicity to normal cancer cells, MCF 10A (IC_50_ > 400 µg/mL) [11]. To this end, the roles of M1S9 in chemoprevention warrant further investigation using in vivo models to mimic the responses of the human body. 

Due to the less tumorigenic nature and slow growth of MCF-7, more aggressive MDA-MB-231 cells were used to induce solid tumors in xenograft mice. At the third week of treatment, the percentage of tumor growth inhibition for high-dose M1S9 and tamoxifen (TAM) was 64.5% (the highest rate throughout the eight weeks) and 69.1%, respectively. TAM-treated mice showed the highest inhibition rate at the sixth week, up to 80.5%. In Ehrlich’s solid tumor implanted mice model (a breast cancer model), high-dose MOL (500 mg/kg) extract was ingested orally for a successive 14 days and exhibited tumor growth inhibition of up to 34.98% [42]. Therefore, we postulated that M1S9 extract possesses better anti-cancer ability in killing the MDA-MB-231 breast cancer cells than MOL extract alone. Moreover, there was no statistically significant difference found in the relative weights of the liver, heart, spleen, lung and kidney organs, indicating the safe usage of M1S9 for up to eight weeks in the current investigation. M1S9 extract exerted growth inhibitory effects in breast cancer cells both in vitro and in vivo. Further delineation of the underlying pathways/mechanisms of action is important to understand the molecular mechanism of action of M1S9 on MDA-MB-231 cell lines. 

**Figure 4:**
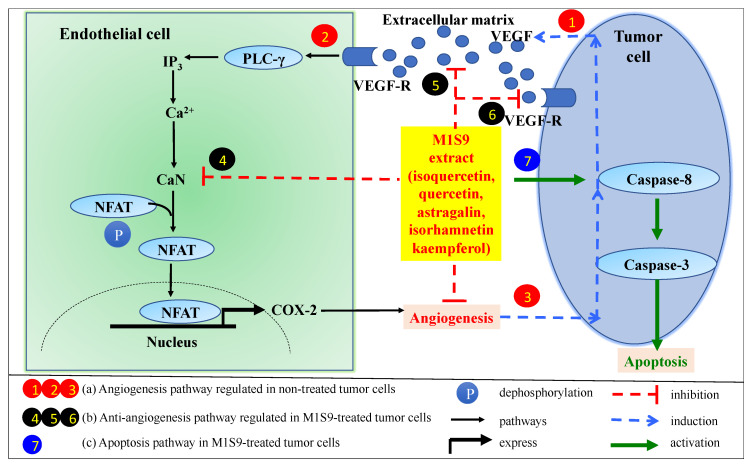
Possible pathways involved in tumorigenesis, angiogenesis and cell apoptosis. (a) Angiogenesis pathway in non-treated tumor cells. (1) Tumor cells secrete VEGF into the extracellular matrix and then bind to VEGFR on endothelial cells to activate the NFAT signaling cascade for VEGF-induced angiogenesis. (2) VEGFR pathway activates phospholipase Cγ (PLCγ) and releases calcium via inositol-1,4,5-triphosphate (IP3) to activate calcineurin (CaN) activity via calmodulin (a calcium-binding protein). Activated CaN regulates NFAT dephosphorylation to enter into nucleus to regulate cyclooxygenase-2 (COX-2) gene expression that can stimulate angiogenesis process. (3) VEGF-mediated angiogenesis induces more VEGF secretion for blood vessel formation. (b) Anti-angiogenesis pathway in M1S9-treated tumor cells. (4) M1S9 extract might inhibit CaN activity without inducing NFAT pathway. As a consequence, it will limit (5) VEGF release from tumor cell and limit (6) the dimer formation of VEGF to VEGF-R. (c) Apoptosis pathway in M1S9-treated tumor cells. (7) M1S9 extract activates caspase-8 in tumor cell that govern the apoptosis pathway (adapted and modified from Zhao et al., 2016).

Angiogenesis is characterized by the formation of blood vessels, which is a critical factor for the cancer cells to spread to the other organs, termed metastases. Vascular endothelial growth factor (VEGF) is an important marker for angiogenesis and acts as the main pro-angiogenic factor in the angiogenesis of breast cancer [43]. M1S9-treated mice showed a significant reduction in VEGF proteins, suggesting that M1S9 might inhibit endothelial cell proliferation or block the effects of VEGF on the endothelial cells. However, further investigation using 5-bromo-2′-deoxy-uridine (BrdU) that labels the newly synthesized DNA in the living cells to identify proliferating cells is warranted to confirm this. Vascular endothelial growth factor receptor 2 (VEGFR2) mediated calcineurin/nuclear factor of activated T-cells (NFAT) pathway plays leading roles in the angiogenesis of breast cancer. Calcineurin (CaN) is a target enzyme in this pathway. In endothelial cells, VEGFR2 activates CaN, triggers NFAT translocated into nucleus and upregulates angiogenic factors [44]. From metabolite profiling, M1S9 extract possesses flavonoid-based compounds, such as isoquercetin, quercetin, astragalin, isorhamnetin and kaempferol. These flavonoid-based compounds have been reported to act as inhibitors of CaN which block the CaN/NFAT pathway (pathway that promotes cancer growth, invasion, migration and angiogenesis) and consequently suppress angiogenesis [44]. Therefore, we hypothesize that flavonoid-based compounds in M1S9 are potential inhibitors of CaN and could probably trigger several pathways, as illustrated in Figure 4, which warrants further confirmation. 

At the molecular level, only the solute carrier family 39, member 6 (SLC39A6) and secreted frizzle-related protein 1 (SFRP1) genes were significantly downregulated in M1S9-treated MDA-MB-231 tumor. SLC39A6, also known as LIV-1, is a zinc transporter involved in the metabolism of protein, nucleic acid, carbohydrate and lipid and also regulates gene transcription, growth, development and differentiation [45]. Deregulated expression of zinc transporters could perturb the regulation of zinc in cellular processes, leading to the initiation or progression of breast cancer [46]. Overexpression of SLC39A6 increased cell proliferation and regulated metastasis in esophageal and cervical cancer cells while knockdown of SLC39A6 suppressed cell proliferation and reduced lymphatic metastasis [47]. These studies together demonstrate that the downregulation of SLC39A6 gene in M1S9-treated MDA-MB-231 tumors could be associated with lower tumor volumes, due to the inhibition of cancer cell proliferation. In the MDA-MB-231 xenograft model, SFRP1 suppressed tumor growth by blocking the Wnt signaling and inhibited cancer cell adhesion and migration by blocking thrombospondin-1 [48,49,50]. Loss-of-function test on SFRP1 in triple negative breast cancer modified the tumorigenic properties of the cells through pro-apoptotic and migratory pathways but did not rely on Wnt signaling. On the other hand, in gastric cancer, overexpressed SFRP1 gene was associated with the activation of transforming growth factor-beta (TGFβ) signaling pathway that induced cell proliferation, epithelial–mesenchymal transition (EMT) and invasion [51]. In other words, downregulation of SFRP1 in M1S9-treated MDA-MB-231 tumors could probably reduce/inhibit tumorigenesis and metastasis, leading to a less aggressive phenotype. M1S9 extract was postulated to exert chemopreventive potential by regulating the apoptotic process, cell cycle, cell death and survival in MDA-MB-231 cancer cells, which requires further research.

In another context, the anti-cancer potential of M1S9 extract can probably be attributed to the glucosinolates which have been hydrolyzed into active metabolite, isothiocyanate (ITC). Hydrolysis of glucosinolates is catalyzed by myrosinase from a plant source in the small intestine or bacterial myrosinase in the colon at neutral pH [52]. There are two types of glucosinolates found in M1S9 extract that are primarily from MOSr, i.e., glucosinalbin and glucotropaeolin compounds, which can be converted into 4-hydroxybenzyl ITC and benzyl ITC, respectively [52]. Benzyl ITC has been previously found to display anti-proliferative, anti-cancer and anti-metastatic properties [53]. Jurkowska et al. (2018) reported that 4-hydroxybenzyl ITC from white mustard seeds possesses an anti-proliferative effect on human neuroblastoma (SH-SY5Y) and glioblastoma (U87MG) [54]. These implied that M1S9 could potentially demonstrate anti-tumor activity with the presence of benzyl ITC and 4-hydroxybenzyl ITC, and its underlying mechanism remains to be elucidated. 

Although M1S9 extract possesses anti-tumor activity, its effectiveness was lower than the TAM-treated model and it was toxic to PMECs. Gong et al. (2018) showed that lutein (a type of carotenoid) inhibited MDA-MB-468 and MCF-7 breast cancer cell growth without affecting the growth of PMECs [55]. This suggests that the removal of certain phytochemical compounds present in M1S9 might improve the treatment efficiency so that PMECs are spared from the toxic effects. 

Several studies have investigated the combined effects of MO extracts with chemotherapeutic drugs. This is because phytochemical compounds in the moringa plant possess an anti-tumor function with low toxicity, low side effects and low tumor resistance to normal cells [8]. The combined effect of ethanolic extract of MO and doxorubicin showed enhanced cytotoxic effect by triggering apoptosis against HeLa cervical cancer cells [56]. In pancreatic cancer cells, aqueous extract of MOL not only inhibited the growth of cancer cells through NF-kB signaling but also improved the efficiency of chemotherapy when combined with cisplatin (a drug for pancreatic cancer) [57]. In breast cancer, the combination of a bioactive subfraction of *Srobilanthes crispus* and tamoxifen demonstrated an anti-cancer response in MCF-7 and MDA-MB-231 cell lines and did not show any cytotoxic side effects of the drug on non-malignant MCF-10A cells [58]. 

In order to achieve better cytotoxic effects with low side effects against normal cells, further study on the different range of ratios for MOL and MOSr extracts would be beneficial to identify a moringa mixture that is enriched with all identified compounds with anti-cancer properties. Compounds which exhibit more profound selectivity against cancer cells in vivo and in vitro, without killing the non-cancer cells, are desired. Moreover, the combined effect of the extracts with tamoxifen also can be investigated to gain insights into whether it will enhance anti-tumor function with low toxicity, low side effects and low tumor resistance to normal cells. 

## 5. Conclusions

Moringa mixture, M1S9, exhibited cytotoxic activities towards PMECs and MDA-MB-231 cell lines which were not related to the antioxidant activity. We hypothesized that M1S9 extract could probably inhibit breast cancer angiogenesis by targeting angiogenic proteins such as VEGF and calcineurin. However, in order to confirm this, further angiogenesis assays including proliferation, migration and tube formation assays are required. M1S9 might exhibit anti-cancer potential by interfering with the signal transduction cascade responsible for cancer proliferation and progression. Postulated signaling pathways (MAPK and ERK1/2 cascade) affected by M1S9 extract can be confirmed using Western blot for ERK1/2 and MAPK markers. Although the mechanism governed by M1S9 extract is not completely delineated at the molecular level, this study is impactful as it provides information about moringa seed residue (MOSr) which can be translated to a usable product for the treatment of aggressive breast cancer. As for this study, both protein levels and differential expression of genes detected served as preliminary information, and further studies are required to confirm the findings. Future studies involving quantitation of more proteins and genes would be required to confirm the modes of action of M1S9 extract. 

## Figures and Tables

**Table 1:** Moringa mixture analysis: (a) qualitative phytochemical analysis; (b) quantitative phytochemical analysis; (c) antioxidant assays (DPPH and Ferric Reducing Antioxidant Power, FRAP) of combined mixture of moringa leaves and seed residue (MOLSr) extracts at different ratios (M1S9, M1S1 and M9S1).

	Control		MOLSr		
	Negative	Positive	M1S9	M1S1	M9S1
(a) Qualitative Phytochemical Analysis					
Tannins	−	++++	+	+++	+++
Triterpenoids	−	++++	−	−	−
Flavonoids	−	++++	−	+	++
Saponins	−	+++	++	−	−
Anthraquinone glycosides	−	++++	−	−	−
Phenols	−	++++	+	+++	++++
Steroids	−	++++	−	−	−
Alkaloids	−	++++	−	−	−
Proteins	−	+	+	+	+
Amino acids	−	++++	+	−	−
Fats and fixed oils	−	++++	+++	++	++
Carbohydrates	−	++++	+++	++	++
**(b) Quantitative Phytochemical Analysis**					
TFC (µg QE/mg)	NR	NR	0.88 ± 0.09	2.58 ± 0.11	4.72 ± 0.06
TPC (µg GAE/mg)	NR	NR	57.7 ± 1.03	68.0 ± 0.53	82.2 ± 4.57
**(c) Antioxidant Assays**					
DPPH (IC_50_, µg/mL)	1.2 ± 0.10 *	−	113.43 ± 25.45	12.50 ± 3.67	11.65 ± 4.99
FRAP (µg Trolox/mg)	−	172.41 ± 12.6 ^$^	1.37 ± 0.56	2.04 ± 0.67	5.60 ± 0.73

(a) For qualitative phytochemical analysis, (−) means the absence of the phytochemical; (+) means the presence of the phytochemical, amount based on the color intensity from lowest to highest, (+) < (++) < (+++) < (++++); (NR) means non-related. (b) For quantitative phytochemical analysis, total flavonoid content (TFC) and total phenolic content (TPC) are presented as mean ±2 standard deviations (n = 3) with units of µg QE/mg (µg of quercetin equivalent per mg extracts) and µg GAE/mg (µg of gallic acid equivalent per mg extracts), respectively. Note: NR—non-relevant. (c) For antioxidant assays, FRAP value was presented as mean ±2 standard deviation (n = 3) with unit of µg Trolox per µg sample dry weight (µg Trolox/mg). Note: *—positive control using Trolox; ^$^—positive control using ascorbic acid.

**Table 2:** List of compounds identified in the moringa leaves (MOL), moringa seed residue (MOSr) and combined mixture of moringa leaves and seed residue (MOLSr) extracts at different ratios (M1S9, M1S1 and M9S1) according to retention time, molecular weight and metabolite classification.

Compound Group	Compound Name	Retention Time	Molecular Formula	Molecular Weight	Percentage of Relative Abundance (%)				
					MOL	MOSr	M1S9	M1S1	M9S1
Glucosinolates	Glucosinalbin	1.351	C_14_H_19_NO_10_S_2_	425.0419	ND	0.107	0.046	0.029	ND
Glucosinolates	Glucotropaeolin	1.395	C_14_H_19_NO_9_S_2_	409.0502	ND	0.016	0.004	ND	ND
Miscellaneous	Cinnamic acid	1.667	C_9_H_8_O_2_	148.0516	0.090	0.020	0.060	0.060	0.090
Miscellaneous	Quinoline	2.501	C_9_H_7_N	129.0575	0.007	ND	0.004	0.005	0.008
Miscellaneous	Benzoic acid	5.913	C_7_H_6_O_2_	122.037	ND	0.010	0.007	ND	ND
Glycoside	Niazirin	8.212	C_14_H_17_NO_5_	279.1121	ND	0.009	0.009	0.006	0.005
Flavonoid	isoquercetin	10.422	C_21_H_20_O_12_	464.0956	1.030	ND	0.630	0.930	1.230
Flavonoid	Quercetin	10.442	C_15_H_10_O_7_	302.0436	0.560	ND	0.350	0.510	0.680
Flavonoid	Astragalin	11.478	C_21_H_20_O_11_	448.1009	0.370	ND	0.240	0.340	0.510
Steroid	Strophanthidin	11.703	C_23_H_32_O_6_	404.2181	0.050	0.01	0.020	0.030	0.040
Flavonoid	Isorhamnetin	11.772	C_16_H_12_O_7_	316.0584	0.040	ND	0.020	0.030	0.040
Flavonoid	Kaempferol	12.291	C_15_H_10_O_6_	286.0479	0.060	ND	0.020	0.040	0.060
Glycoside	Niazimicin	14.802	C_16_H_23_NO_6_S	357.1252	0.050	ND	0.030	0.040	0.050
Phenolic acid	p-coumaric acid	20.79	C_9_H_8_O_3_	164.0475	ND	0.005	0.005	ND	ND
Miscellaneous	Vanillin	15.878	C_8_H_8_O_3_	152.0479	ND	0.010	0.008	ND	ND
Miscellaneous	Heptadecane	26.47	C_17_H_36_	240.2828	ND	0.003	ND	ND	0.002
Miscellaneous	Palmitic acid	34.17	C_16_H_32_O_2_	256.2414	0.004	ND	ND	0.003	0.003

Note: ND—non-determined.

**Table 3:** The IC_50_ values of tamoxifen (TAM) (positive control) and combined mixture of moringa leaves and seed residue (MOLSr) extracts at different ratios (M1S9, M1S1 and M9S1) after 24, 48 and 72 h of treatment with respective cell lines.

Cell Lines	Time Points (h)	IC_50_ (μg/mL)			
		TAM	M1S9	M1S1	M9S1
Primary mammary epithelial cells (PMECs)	24	4.0	70.0	82.0	>100.0
	48	4.0	33.5	78.5	>100.0
	72	3.0	17.5	51.0	>100.0
Non-malignant Chang’s liver cells	24	4.0	>100.0	>100.0	>100.0
	48	6.0	93.5	>100.0	>100.0
	72	3.0	>100.0	>100.0	>100.0
Hepatocellular carcinoma (HepG2)	24	7.0	>100.0	>100.0	>100.0
	48	4.0	>100.0	>100.0	>100.0
	72	3.0	>100.0	>100.0	>100.0
Colorectal carcinoma cells (HCT-116)	24	9.0	>100.0	>100.0	>100.0
	48	5.5	>100.0	>100.0	>100.0
	72	4.5	>100.0	>100.0	>100.0
Cervical adenocarcinoma cells (HeLa)	24	5.0	91.0	>100.0	>100.0
	48	3.5	75.0	>100.0	>100.0
	72	3.0	81.0	91.0	>100.0
Breast adenocarcinoma cells (MCF-7)	24	15.5	97.5	>100.0	>100.0
	48	7.5	53.0	>100.0	>100.0
	72	8.0	38.5	57.0	>100.0

## Supplementary Materials

The following are available online at https://www.mdpi.com/2072-6643/12/10/2993/s1, Figure S1: Experimental design, Figure S2: General methodology of in vivo xenograft mice model, Figure S3: Metabolite profiles of (a) moringa leaves (MOL); (b) combined mixture of moringa leaves (MOL) and seed residue (MOSr) at different ratios; (c) M1S9; (d) M1S1; (e) M1S9, Table S1: Functional grouping of differential expressed genes following the treatment with tamoxifen (TAM) and M1S9, Table S2: Gene ontology (GO) terms for upregulated and downregulated genes (≥2-fold) in (a) TAM-treated versus non-treated group, Table S3: Gene ontology (GO) terms for upregulated and downregulated genes (≥2-fold) in (b) M1S9-treated versus non-treated group, Table S4: Pathway analysis for upregulated and downregulated genes (≥2-fold) in (a) TAM-treated versus non-treated group and (b) M1S9-treated versus non-treated group, Table S5: Top three enriched networks and their associated molecules, score and focus molecules in (a) TAM-treated and (b) M1S9-treated MDA-MB-231 xenograft tumor.
