# Supplementary material for: Significant Decreased Expressions of CaN, VEGF, SLC39A6 and SFRP1 in MDA-MB-231 Xenograft Breast Tumor Mice Treated with Moringa oleifera Leaves and Seed Residue (MOLSr) Extracts
Source: Nutrients. 2020 Sep 30;12(10):2993. doi: 10.3390/nu12102993 (PMC7601446; doi:10.3390/nu12102993)
Supplement: Supplementary file 1 [file nutrients-12-02993-s001.pdf]

Supplementary material

Figure S1. Experimental design. Firstly, phytochemical screening, antioxidant test and metabolite profiling were carried out on the combined effect of *Moringa oleifera* leaves and seed residue (MOLSr) extracts. *In vitro* assay was carried on primary mammary epithelial cells (PMEC) and various human cancer cell lines to assess the cell viability using MTT assay. The most potent MOLSr was chosen for establishment of *in vivo* xenograft mice model. Profound tumour growth inhibition and specific concentration of M1S9 was used for protein and gene expression analysis, using ELISA assay and PCR array, respectively.

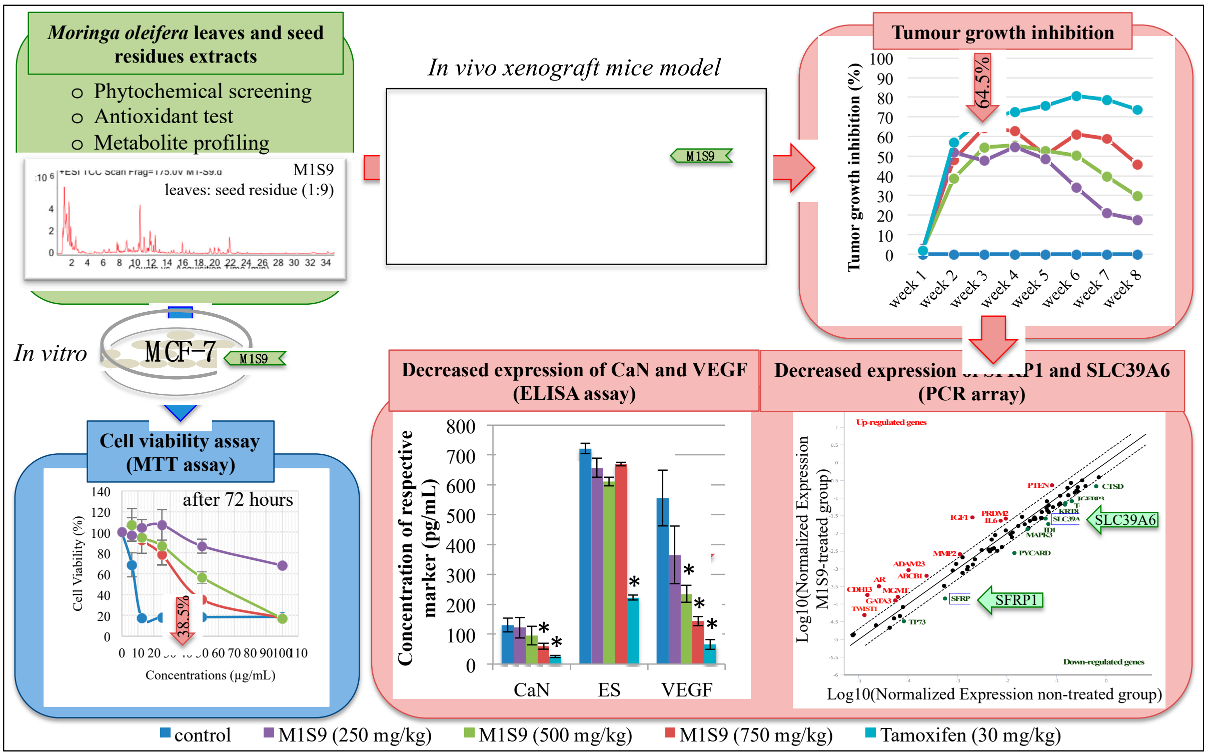

Figure S2. General methodology of *in vivo* xenograft mice model. Tumour-bearing micewith tumour that reached a volume of 100–150 mm^3 were selected to receive combined mixture of moringa leaves and seed residue (MOLSr) extract (i.e. M1S9) at different dosages (250 mg/kg, 500 mg/kg and 750 mg/kg), normal saline (control negative) and positive control (tamoxifen (30 mg/kg). Moringa extracts and normal saline were administered orally, whereas tamoxifen was injected subcutaneously. All treatments were given on daily basis for

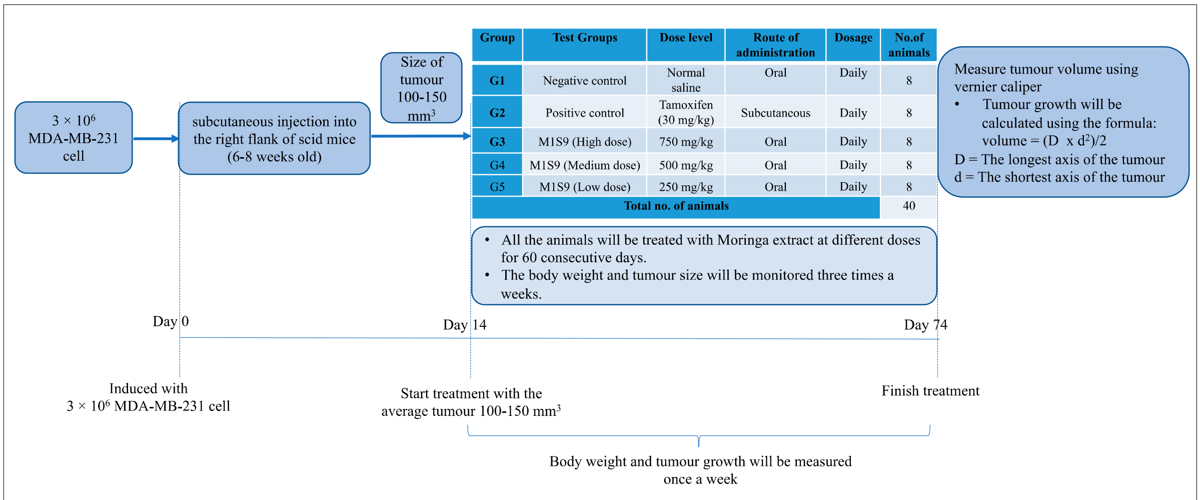

Note: M1S9 extract consisted of 1 mg of Moringa leave (MOL) extract and 9 mg of Moringa seed residue (MOSr) extract.

Figure S3. Metabolite profiles of (a) moringa leaves (MOL), (b) combined mixture of moringa leaves (MOL) and seed residue (MOSr) at different ratios: (c) M1S9, (d) M1S1 and (e) M1S9. The peaks show the bioactive compounds in the respective chromatograms at positive ESI ionization mode.

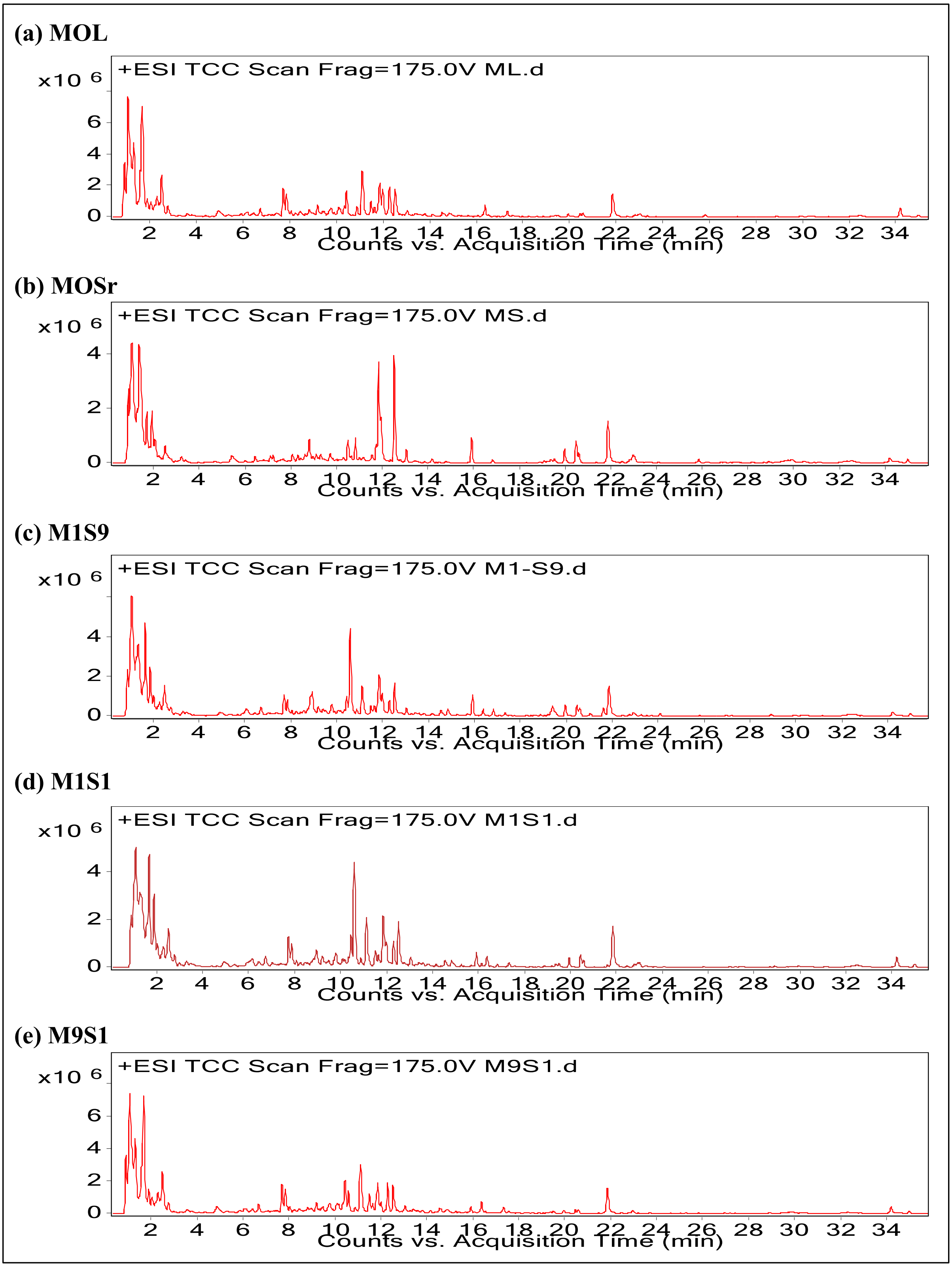

Table S1. Functional grouping of differentially expressed genes following the treatment with tamoxifen (TAM) and M1S9. Gene names with bold and asterisk (*) are up/down-regulated significantly at *p*-value less than 0.05. Abbreviation: BCCM-breast cancer classification markers; BC-breast cancers; ST-signal transduction.

**Classification**	**(a) TAM-treated versus non-treated group**		**(b) M1S9-treated versus non-treated group**		**(c) M1S9-treated versus TAM-treated group**	
	**Up-regulated**	**Down-regulated**	**Up-regulated**	**Down-regulated**	**Up-regulated**	**Down-regulated**
**BCCM (Luminal A-C BC)**	**ESR1***, GATA3, TFF3	-	GATA3	KRT8, **SLC39A6***	-	ESR1, SLC39A6, TFF3
**BCCM (HER2-Like BC)**	-	-	-	-	-	-
**BCCM (Basal-Like/Triple Negative BC)**	KRT5	-	-	-	-	BIRC5, EGFR, KRT5
**BCCM (BC Metastasis to Lung)**	-	ID1	MMP2	ID1	-	-
**ST (Steroid Receptor-Mediated Signalling)**	**AR*, ESR1***	**CTNNB1***	AR, IGF1	-	CTNNB1, IGF1	BRCA1, ESR1, KRT19, **RB1***
**ST (Hedgehog Signalling)**	-	-	-	-	-	-
**ST (Glucocorticoid Signalling)**	**NR3C1***	-	-	IGFBP3	-	IGFBP3, **NME1***, NR3C1
**ST (Classical WNT Signalling)**	-	**CTNNB1***	-	**SFRP1***	CTNNB1	APC, SFRP1
**ST (AKT & PI3 Kinase Signalling)**	-	-	IGF1, PTEN	-	IGF1, PTEN	AKT1
**ST (Notch Signalling)**	-	-	-	-	-	BIRC5
**ST (MAP Kinase Signalling)**	MAPK8	-	-	MAPK3, TP73	-	MAPK3, MAPK8, TP73
**Epithelial-to-mesenchymal transition (EMT)**	TWIST1	**CTNNB1***	TWIST1	TGFB1	CTNNB1	-
**Angiogenesis**	CDH13, EGF, **IL6***, SLIT2, **THBS1***	**CTNNB1***, ID1	CDH13, IL6, PTEN	ID1	CTNNB1, PTEN	EGF, THBS1
**Cell Adhesion Molecules**	ADAM23, CDH1, CDH13, CDKN2A, **THBS1***	**CTNNB1***	ADAM23, CDH13, PTEN	TGFB1	ADAM23, CTNNB1, PTEN	APC, CDH1, CDKN2A, EGFR, THBS1
**Proteases**	ADAM23	CTSD	ADAM23, MMP2	CTSD, PYCARD	ADAM23	**PYCARD***
**Apoptosis**	CDH1, CDKN2A, **IL6***, TWIST1		IGF1, IL6, TWIST1	**SFRP1***, TP73	IGF1	AKT1, APC, CDH1, **CDKN1A***, CDKN2A, **NME1***, SFRP1, TP73
**Cell Cycle**	CCNA1, CCND2, CDKN1C, CDKN2A, **MYC***	RASSF1	PTEN	-	PTEN, RASSF1	APC, CCNA1, **CDKN1A***, CDKN1C, CDKN2A, MKI67, **MYC***, **RB1***
**DNA Damage & Repair**			MGMT	TP73		APC, ATM, BRCA1, BRCA2, **CDKN1A***, **MLH1***, TP73
**Xenobiotic Transport**	ABCB1	-	ABCB1	-	-	-
**Transcription Factors**	**AR*, ESR1***, GATA3, **MYC***, **NR3C1***, PRDM2	**CTNNB1***	AR, GATA3, PRDM2	TP73	CTNNB1	ESR1, **MYC***, NR3C1, **RB1***, TP73
**No. of genes (genes*)**	22 (**6***)	4 (**1***)	12	10 (**2***)	5	31 (**6***)

Table S2. Gene ontology (GO) terms for up-regulated and down-regulated genes (≥2-fold) in (a) TAM-treated versus non-treated group. No GO term for down-regulated genes in group (a) as the gene count less than 3. Genes with bold and asterisk (*) indicate genes with significant difference at $p<0.05$. Abbreviation: *P*-*p*-value; FE-fold enrichment; TAM-tamoxifen.

GO terms	Count	Genes	*p*	FE
**(a) TAM-treated versus non-treated group**				
		Up-regulated genes [n=22, (6*)]		
Positive regulation of transcription, DNA-templated	9	CDKN1C, **AR***, **IL6***, CDKN2A, GATA3, **ESR1***, CDH1, EGF, **MYC***	1.061E-07	13.34
Positive regulation of transcription from RNA polymerase II promoter	9	CDH13, **AR***, **IL6***, CDKN2A, GATA3, **ESR1***, **NR3C1***, **MYC***, TWIST1	1.355E-05	7.00
Response to drug	6	**IL6***, GATA3, CDH1, ABCB1, **THBS1***, **MYC***	3.018E-05	15.06
Negative regulation of apoptotic process	6	**IL6***, CCND2, MAPK8, **THBS1***, **MYC***, TWIST1	2.030E-04	10.07
Positive regulation of cell proliferation	6	**AR***, **IL6***, CCND2, **THBS1***, EGF, **MYC***	2.269E-04	9.83
Transcription, DNA-templated	6	**AR***, CDKN2A, **ESR1***, PRDM2, **NR3C1***, TWIST1	8.869E-02	2.34
Positive regulation of gene expression	5	**AR***, **IL6***, MAPK8, **MYC***, TWIST1	2.812E-04	14.57
Negative regulation of cell proliferation	5	CDH13, **AR***, **IL6***, CDKN2A, GATA3	1.326E-03	9.64
Negative regulation of transcription from RNA polymerase II promoter	5	CDKN1C, GATA3, **ESR1***, **MYC***, TWIST1	1.120E-02	5.30
Signal transduction	5	**AR***, GATA3, **ESR1***, **NR3C1***, EGF	5.296E-02	3.29
Positive regulation of smooth muscle cell proliferation	4	CDH13, **IL6***, **THBS1***, **MYC***	5.510E-05	50.88
Cell cycle arrest	4	CDKN1C, CDKN2A, **THBS1***, **MYC***	6.899E-04	21.65
In utero embryonic development	4	**AR***, GATA3, **MYC***, TWIST1	1.559E-03	16.33
Positive regulation of apoptotic process	4	**IL6***, CDKN2A, MAPK8, SLIT2	5.916E-03	10.18
Negative regulation of transcription, DNA-templated	4	CDKN1C, CDKN2A, GATA3, TWIST1	2.330E-02	6.12
Transcription from RNA polymerase II promoter	4	GATA3, **ESR1***, **NR3C1***, **MYC***	2.504E-02	5.95
Uterus development	3	CDKN1C, GATA3, **ESR1***	1.343E-04	163.56
Mammary gland alveolus development	3	**AR***, **ESR1***, EGF	2.003E-04	134.70
Positive regulation of phosphorylation	3	**AR***, **THBS1***, EGF	4.392E-04	91.59
Positive regulation of epithelial cell proliferation	3	**IL6***, **MYC***, TWIST1	2.524E-03	38.16
Positive regulation of protein kinase B signalling	3	**IL6***, GATA3, **THBS1***	4.882E-03	27.26
Chromatin remodeling	3	GATA3, **ESR1***, **MYC***	5.111E-03	26.63
Cellular response to tumour necrosis factor	3	**IL6***, GATA3, **THBS1***	8.233E-03	20.82
Transcription initiation from RNA polymerase II promoter	3	**AR***, **ESR1***, **NR3C1***	1.527E-02	15.06
Cell division	3	CCND2, **NR3C1***, CCNA1	7.013E-02	6.54

Table S3. Gene ontology (GO) terms for up-regulated and down-regulated genes (≥2-fold) in (b) M1S9-treated versus non-treated group. Genes with bold and asterisk (*) indicate genes with significant different at *p*<0.05. Abbreviation: *P*-*p*-value; FE-fold enrichment; TAM-tamoxifen.

GO terms	Count	Genes	*p*	FE
**(b) M1S9-treated versus non-treated group**				
		Up-regulated genes (n=12)		
Positive regulation of transcription from RNA polymerase II promoter	6	CDH13, AR, IL6, GATA3, IGF1, TWIST1	2.314E-04	8.56
Response to drug	5	IL6, GATA3, MGMT, ABCB1, PTEN	3.144E-05	23.02
Negative regulation of cell proliferation	5	CDH13, AR, IL6, GATA3, PTEN	8.815E-05	17.67
Negative regulation of apoptotic process	5	IL6, MGMT, IGF1, PTEN, TWIST	1.509E-04	15.38
Positive regulation of cell proliferation	4	AR, IL6, IGF1, PTEN	2.967E-03	12.01
Positive regulation of transcription, DNA-templated	4	AR, IL6, GATA3, IGF1	3.937E-03	10.87
Positive regulation of transcription regulatory region DNA binding	3	GATA3, IGF1, TWIST1	4.077E-05	279.87
Positive regulation of epithelial cell proliferation	3	IL6, IGF1, TWIST1	6.764E-04	69.97
Positive regulation of smooth muscle cell proliferation	3	CDH13, IL6, IGF1	6.764E-04	69.97
Positive regulation of MAPK cascade	3	AR, IL6, IGF1	1.229E-03	51.83
Response to ethanol	3	GATA3, MGMT, PTEN	2.053E-03	39.98
In utero embryonic development	3	AR, GATA3, TWIST1	6.351E-03	22.45
Positive regulation of gene expression	3	AR, IL6, TWIST1	1.216E-02	16.02
Cell proliferation	3	AR, IGF1, PTEN	2.288E-02	11.47
		Down-regulated genes [n=10, (2*)]		
Positive regulation of apoptotic process	5	**SFRP1***, PYCARD, IGFBP3, TGFB1, TP73	1.172E-05	27.99
Negative regulation of cell proliferation	4	**SFRP1***, IGFBP3, TGFB1, TP73	9.836E-04	16.96
Positive regulation of transcription, DNA-templated	4	**SFRP1***, MAPK3, TGFB1, TP73	2.098E-03	13.04
Apoptotic process	4	MAPK3, PYCARD, IGFBP3, TP73	2.762E-03	11.85
Positive regulation of epithelial cell proliferation	3	**SFRP1***, ID1, TGFB1	4.448E-04	83.96
Positive regulation of ERK1 and ERK2 cascade	3	MAPK3, PYCARD, TGFB1	3.705E-03	28.79
Regulation of apoptotic process	3	IGFBP3, TGFB1, TP73	5.437E-03	23.65
Viral process	3	KRT8, MAPK3, TP73	1.047E-02	16.85
Response to drug	3	**SFRP1***, TGFB1, TP73	1.081E-02	16.57
Protein phosphorylation	3	MAPK3, IGFBP3, TGFB1	2.334E-02	11.05
Negative regulation of transcription, DNA-templated	3	**SFRP1***, ID1, TGFB1	2.763E-02	10.10
Negative regulation of transcription from RNA polymerase II promoter	3	ID1, TGFB1, TP73	5.410E-02	7.00
Positive regulation of transcription from RNA polymerase II promoter	3	MAPK3, TGFB1, TP73	9.340E-02	5.14

Table S4. Pathway analysis for up-regulated and down-regulated genes (≥2-fold) in (a) TAM-treated versus non-treated group and (b) M1S9-treated versus non-treated group. Asterisk (*) indicate genes with significant different at $p<0.05$. Abbreviation: *P*-*p*-value; FE-fold enrichment; TAM-tamoxifen.

**Pathway**	**Count**	**Genes**	***p***	**FE**
**(a) TAM-treated versus non-treated group**				
		Up-regulated genes [n=22, (6*)]		
Pathways in cancer	7	**AR***, **IL6***, CDKN2A, CDH1, MAPK8, EGF, **MYC***	1.077E-04	7.66
Bladder cancer	5	CDKN2A, CDH1, **THBS1***, EGF, **MYC***	1.414E-06	52.43
				17.34
Cell cycle	5	CDKN1C, CDKN2A, CCND2, CCNA1, **MYC***	1.177E-04	
MicroRNAs in cancer	5	CDKN2A, CCND2, ABCB1, **THBS1***, **MYC***	2.776E-03	7.52
PI3K-Akt signalling pathway	5	**IL6***, CCND2, **THBS1***, EGF, **MYC***	5.462E-03	6.23
FoxO signalling pathway	4	**IL6***, CCND2, MAPK8, EGF	2.770E-03	12.83
Hepatitis B	4	**IL6***, MAPK8, CCNA1, **MYC***	3.466E-03	11.86
Proteoglycans in cancer	4	**ESR1***, **THBS1***, **MYC***, TWIST1	8.508E-03	8.60
Focal adhesion	4	CCND2, MAPK8, **THBS1***, EGF	9.228E-03	8.35
HTLV-I infection	4	**IL6***, CDKN2A, CCND2, **MYC***	1.628E-02	6.77
Endometrial cancer	3	CDH1, EGF, **MYC***	5.526E-03	24.80
Pancreatic cancer	3	CDKN2A, MAPK8, EGF	8.528E-03	19.84
p53 signalling pathway	3	CDKN2A, CCND2, **THBS1***	9.042E-03	19.25
Prolactin signalling pathway	3	CCND2, **ESR1***, MAPK8	1.011E-02	18.17
Melanoma	3	CDKN2A, CDH1, EGF	1.011E-02	18.17
ErbB signalling pathway	3	MAPK8, EGF, **MYC***	1.492E-02	14.83
Epstein-Barr virus infection	3	MAPK8, CCNA1, **MYC***	2.817E-02	10.57
Wnt signalling pathway	3	CCND2, MAPK8, **MYC***	3.537E-02	9.35
Jak-STAT signalling pathway	3	**IL6***, CCND2, **MYC***	3.872E-02	8.90
Hippo signalling pathway	3	CCND2, CDH1, **MYC***	4.169E-02	8.54
Transcriptional misregulation in cancer	3	**IL6***, CCND2, **MYC***	5.001E-02	7.72
Viral carcinogenesis	3	CDKN2A, CCND2, CCNA1	7.195E-02	6.29
Rap1 signalling pathway	3	CDH1, **THBS1***, EGF	7.504E-02	6.14
		Down-regulated genes [n=4, (1*)]		
Hippo signalling pathway	3	ID1, RASSF1, **CTNNB1***	0.001	34.17
**(b) M1S9-treated versus non-treated group**				
		Up-regulated genes (n=12)		
Pathways in cancer	5	AR, IL6, IGF1, PTEN, MMP2	1.444E-04	12.50
Prostate cancer	3	AR, IGF1, PTEN	2.347E-03	33.50
FoxO signalling pathway	3	IL6, IGF1, PTEN	5.367E-03	22.00
Proteoglycans in cancer	3	IGF1, MMP2, TWIST1	1.168E-02	14.74
PI3K-Akt signalling pathway	3	IL6, IGF1, PTEN	3.289E-02	8.55
		Down-regulated genes [n=10, (2*)]		
TGF-beta signalling pathway	3	ID1, MAPK3, TGFB1	2.974E-03	30.71
Hippo signalling pathway	3	ID1, TGFB1, TP73	9.350E-03	17.08
Tuberculosis	3	MAPK3, CTSD, TGFB1	1.270E-02	14.57

Table S5. Top three enriched networks and their associated molecules, score and focus molecules in (a) TAM-treated and (b) M1S9-treated MDA-MB-231 xenograft tumour. Up/down-regulated genes are written in red and green, respectively. Asterisk (*) indicate genes with significant different at $p<0.05$. Abbreviation: TAM-tamoxifen.

Treat-ment	ID	Molecules in Network	Score	Focus Molecules	Top Diseases and Functions
**(a) TAM**	1	APC(complex), BMP, C/EBP, **CCNA1**, **CCND2**, Cdk, **CDKN1C**, **CDKN2A**, CollagenAlpha1, Ctbp, CyclinA, CyclinB, CyclinD, CyclinE, E2f, ERK1/2, Growth hormone, Hedgehog, Hif1, **ID1**, **KRT5**, Nuclear factor1, PEPCK, Pkg, **RASSF1**, Rb, Secretase gamma, Smad, Smad2/3, TCF, TCF/LEF, **TFF3**, TSH, Ubiquitin, Wnt	18	8	[Cancer, Cell Cycle, Connective Tissue Development and Function]
	2	**ABCB1**, Actin, Akt, Alp, Alpha catenin, Ap2, Cadherin, **CDH1**, **CDH13,** Ciap, Collagen type I, **CTSD**, DNA-methyltransferase, Dynamin, estrogen receptor, F Actin, Fgf, Fgfr, FKHR, Foxo, Hsp27, JUN/JUNB/JUND, Laminin1, N-Cadherin, Ngf, Notch, P glycoprotein, PI3K p85, Pld, **PRDM2**, Rock, **SLIT2**, trypsin, TWIST, **TWIST1**	15	7	[Cancer, Cell Morphology, Cellular Movement]
	3	26s Proteasome, **ADAM23**, Alpha tubulin, AMPK, **AR***, caspase, Cbp/p300, CYP, cytochrome C, **GATA3**, GNRH, Hdac, hemoglobin, HISTONE, histone deacetylase, Histone h3, Histone h4, HSP, Hsp70, Hsp90, IgG, Igg3, **IL6***, Interferon alpha, N-cor, **NR3C1***, PARP, PTK, RNA polymerase II, STAT5a/b, TCR, TFIIH, TH2 Cytokine, thymidine kinase, Tlr	10	5	[Cancer, Organismal Injury and Abnormalities, Reproductive System Disease]
**(b) M1S9**	1	7S NGF, Akt, Alpha tubulin, C/EBP, caspase, Cdc2, **CDH13**, Cyclin A, Cyclin E, E2f, estrogen receptor, Foxo, **GATA3**, Growth hormone, Hdac, Histone h4, Hsp90, **ID1**, **IGF1**, Igfbp, **IGFBP3**, JUN/JUNB/JUND, MAP2K1/2, **MGMT**, N-cor, Notch, Nr1h, PARP, PP2A, **PRDM2**, Raf, Rb, Rxr, SAA, TSH	16	7	[Cell Cycle, Cell Death and Survival, Cell Morphology]
	2	26s Proteasome, Actin, **ADAM23**, ADCY, Alpha 1 antitrypsin, Alpha catenin, AMPK, calpain, CG, Creb, **CTSD**, Cyclin B, cytochrome C, cytochrome-c oxidase, Fgf, FSH, GNRH, Gsk3, **IL6**, Integrin, **KRT8**, Lh, **MAPK3**, MTORC1, p70 S6k, PI3K (complex), Pkc(s), PRKAA, **PTEN**, PTK, TCF, TCR, **TP73**, trypsin, Vegf	16	7	[Cellular Growth and Proliferation, Hematological System Development and Function, Immunological Disease]
	3	Alp, collagen, Collagen Alpha1, Collagen type I, Collagen type II, Collagen type III, Collagen type IV, Collagen(s), Cyclin D, ERK1/2, Fc gamma receptor, Fcer1, Fibrin, Hif1, Hsp27, Integrin alpha V beta 3, Laminin (complex), Laminin1, **MMP2**, NADPH oxidase, Pdgf (complex), Pdgf Ab, PDGF BB, PDGF-AA, PLA2, Rock, **SFRP1***, **SLC39A6***, SMAD1/5, Sphk, SYK/ZAP, Tgf beta, Timp, **TWIST1**, Wnt	8	4	[Connective Tissue Development and Function, Skeletal and Muscular System Development and Function, Tissue Development]
